# Uric acid: A new look at an old risk marker for cardiovascular disease, metabolic syndrome, and type 2 diabetes mellitus: The urate redox shuttle

**DOI:** 10.1186/1743-7075-1-10

**Published:** 2004-10-19

**Authors:** Melvin R Hayden, Suresh C Tyagi

**Affiliations:** 1 Department of Family and Community Medicine, University of Missouri, Columbia, Missouri USA; 2 Department of Physiology and Biophysics, University of Louisville, School of Medicine, Louisville, Kentucky USA

## Abstract

**Background:**

The topical role of uric acid and its relation to cardiovascular disease, renal disease, and hypertension is rapidly evolving. Its important role both historically and currently in the clinical clustering phenomenon of the metabolic syndrome (MS), type 2 diabetes mellitus (T2DM), atheroscleropathy, and non-diabetic atherosclerosis is of great importance.

**Results:**

Uric acid is a marker of risk and it remains controversial as to its importance as a risk factor (causative role). In this review we will attempt to justify its important role as one of the many risk factors in the development of accelerated atherosclerosis and discuss its importance of being one of the multiple injurious stimuli to the endothelium, the arterial vessel wall, and capillaries. The role of uric acid, oxidative – redox stress, reactive oxygen species, and decreased endothelial nitric oxide and endothelial dysfunction cannot be over emphasized.

In the atherosclerotic prooxidative environmental milieu the original antioxidant properties of uric acid paradoxically becomes prooxidant, thus contributing to the oxidation of lipoproteins within atherosclerotic plaques, regardless of their origins in the MS, T2DM, accelerated atherosclerosis (atheroscleropathy), or non-diabetic vulnerable atherosclerotic plaques. In this milieu there exists an antioxidant – prooxidant urate redox shuttle.

**Conclusion:**

Elevations of uric acid > 4 mg/dl should be considered a "red flag" in those patients at risk for cardiovascular disease and should alert the clinician to strive to utilize a global risk reduction program in a team effort to reduce the complications of the atherogenic process resulting in the morbid – mortal outcomes of cardiovascular disease.

## Background

While the topicality of serum uric acid (SUA) being a risk factor is currently controversial [1,2], there is little controversy regarding its association as a risk marker associated with cardiovascular (CVD) and renal disease (especially in patients with hypertension, diabetes, and heart failure). SUA seems to be a graded marker of risk for the development of coronary heart disease (CHD) or cerebrovascular disease and stroke compared with patients with normal uric acid levels and especially those in the lower 1/3 of its normal physiological range [1,3-13].

LK Niskanen's *et al*. recently published article has demonstrated new information regarding this subject. They were able to demonstrate that elevations of SUA levels were independent of variables commonly associated with gout or the metabolic syndrome in association with CVD mortality in middle aged men [3].

In 1951, Gertler MM and White PD *et al*. sat out to determine the clinical aspects of premature coronary heart disease in 100 male patients 40 years old and younger. Their findings were increased mesomorphic body build, shorter stature, increased anterior posterior chest wall diameter, and increased cholesterol and uric acid (5.13 +/- .11 vs. 4.64 +/-.06) as compared to the normal population [14].

A much larger trial (1967) confirmed the initial interest in SUA and CVD with the publication of the early, large (5,127 participants), epidemiologic, seminal Framingham study. This classical paper by Kannel *et al*. noted an elevated SUA was also associated with an increased risk of coronary heart disease for men aged 30–59 [15]. In addition to the important finding of elevations in lipoproteins (specifically cholesterol levels greater than 250 mg/100 ml) being associated with CHD, there also appeared a definite association of elevated SUA, which was associated with an increase in the incidence rate of CHD. The above authors also noted that subjects in this study with evidence of impaired carbohydrate metabolism or disordered purine metabolism could be assumed to have accelerated atherogenesis [15].

This controversy regarding SUA being a risk factor or a risk marker is not as important as understanding its overall role in the association with endothelial cell damage, dysfunction, decreased endothelial nitric oxide (eNO) bioavailability, and how SUA interacts with other substrate toxicities and increased reactive oxygen species (ROS) of the A-FLIGHT-U acronym, which result in accelerated atherosclerosis (table 1). Johnson RJ *et al. *have nicely demonstrated that hyperuricemia predicts cardiovascular events in the general population, the hypertensive population, and patients with pre-existing CVD. Furthermore hyperuricemia predicts the development of future hypertension [11].

**Table 1:** A-FLIGHT-U ACRONYM Identification of multiple metabolic toxicities and injurious stimuli responsible for reactive oxygen species production. (figure 2)

**A**	Angiotensin II (also induces PKC-β isoform) Amylin (hyperamylinemia) / amyloid toxicity AGEs/AFEs (advanced glycosylation/fructosylation endproducts) Apolipoprotein B Antioxidant reserve compromised Absence of antioxidant network Aging ADMA (Asymmetrical DiMethyl Arginine)
**F**	Free fatty acid toxicity: **Obesity toxicity: Triad**
**L**	Lipotoxicity – Hyperlipidemia – **Obesity toxicity: Triad**
**I**	Insulin toxicity (endogenous hyperinsulinemia-hyperproinsulinemia) Inflammation toxicity
**G**	Glucotoxicity (compounds peripheral insulin resistance) reductive stress Sorbitol/polyol pathway Pseudohypoxia (increased NADH/NAD ratio)
**H**	Hypertension toxicity Homocysteine toxicity hs-CRP
**T**	Triglyceride toxicity: **Obesity toxicity: Triad**
**U**	**Uric Acid toxicity:**Antioxidant early in physiological range and a conditional prooxidant late when elevated through the paradoxical (antioxidant → prooxidant)
	**URATE REDOX SHUTTLE**
	Endothelial cell dysfunction with eNOS uncoupling, decreased eNO and increased ROS.
	**Vulnerable atherosclerotic plaque milieu of being acidic, proinflammatory, excess metal ions (Fe) (Cu) from vasa vasorum rupture and red blood cell plasma membranes due to intraplaque hemorrhage and plaque thrombus formation.**

There are certain clinical clustering groups with increased cardiovascular risk, which have associated hyperuricemia (table 2). Non-diabetic patient groups with accelerated atherosclerosis, T2DM patient groups with accelerated atherosclerosis (atheroscleropathy), congestive heart failure patient groups with ischemic cardiomyopathy, metabolic syndrome patient groups (with hyperinsulinemia, hypertension, dyslipidemia, impaired glucose tolerance, and obesity), renal disease patient groups, hypertensive patient groups, African American patient groups, patient groups taking diuretics, and patient groups with excessive alcohol usage. Each of these clustering groups has metabolic mechanisms that may help to explain why SUA may be elevated (table 2). In addition to the recurring finding of an elevated tension of oxidative- redox stress and ROS in many of the groups is the importance of the MS and insulin resistance.

**Table 2:** Hyperuricemia: clinical clusters at cardiovascular risk

GROUPS	Abbreviated Mechanisms
Patients with CVD Accelerated atherosclerosis Congestive heart failure	Increased apoptosis – necrosis of the arterial vessel wall and capillary resulting in increased purine metabolism and hyperuricemia. Increased oxidative – redox stress Antioxidant – Prooxidant Paradox: Urate Redox Shuttle
Patients with (T2DM) Accelerated atherosclerosis (Atheroscleropathy)	Acting through obesity and insulin resistance. Accelerated atherosclerosis with increased vascular cell apoptosis and inflammatory necrosis with increased purine metabolism resulting in hyperuricemia and increased oxidative stress through ischemia-reperfusion and xanthine oxidase. Additional reductive stress associated with glucotoxicity and pseudohypoxia. Increased oxidative-redox stress Antioxidant – Prooxidant Paradox: Urate Redox Shuttle
Obesity – Insulin resistance Hyperinsulinemia – Insulin toxicity Metabolic Syndrome (figure 1): Hyperinsulinemia Hypertension Hyperlipidemia dyslipidemia, obesity Hyperglycemia	Leptin may induce hyperuricemia. Insulin increases sodium reabsorption and is tightly linked to urate reabsorption. Increased oxidative – redox stress Antioxidant – Prooxidant Paradox: Urate Redox Shuttle
Men and Postmenopausal females	Estrogen is uricosuric
Renal diseases	Decreases in GFR increases uric acid levels
Hypertension	Urate reabsorption increased in setting of increased renal vascular resistance, microvascular disease predisposes to tissue ischemia that leads to increased urate generation (excess purine metabolism) and reduced excretion (due to lactate competing with urate transporter in the proximal tubule). Increased oxidative – redox stress Antioxidant – Prooxidant Paradox: Urate Redox Shuttle
African American	Unknown (assumed genetic causes as yet unidentified)
Diuretic use	Volume contraction promotes urate reabsorption
Alcohol use (in excess)	Increases urate generation and decreased urate excretion

## Uric acid, MS, T2DM, and atheroscleropathy

The importance of hyperuricemia and the clustering phenomenon of the metabolic syndrome were first described by Kylin in 1923 when he described the clustering of three clinical syndromes: hypertension, hyperglycemia, and hyperuricemia [16]. In 1988, Reaven GM described the important central role of insulin resistance in the seminal Banting lecture where he described Syndrome X, which has now become known as the metabolic syndrome (MS) and/or the insulin resistance syndrome (IRS) [17]. Seven decades after the clustering phenomenon was reported by Kylin (1993), Reaven GM and Zavaroni I *et al. *suggested that hyperuricemia be added to the cluster of metabolic and hemodynamic abnormalities associated with insulin resistance and/or hyperinsulinemia of Syndrome X [18].

The four major players in the MS are hyperinsulinemia, hypertension, hyperlipidemia, and hyperglycemia. Each member of this deadly quartet has been demonstrated to be an independent risk factor for CHD and capable of working together in a synergistic manner to accelerate both non-diabetic atherosclerosis and the atheroscleropathy associated with MS, PD, and T2DM.

In a like manner, hyperuricemia, hyperhomocysteinemia, ROS, and highly sensitive C- reactive protein (hsCRP) each play an important role in expanding the original Syndrome X described by Reaven in the atherosclerotic process. The above quartet does not stand alone but interacts in a synergistic manner resulting in the progression of accelerated atherosclerosis and arterial vessel wall remodeling along with the original players and the A-FLIGHT-U toxicities (table 1). The MS of clinical clustering has been renamed multiple times over the past 16 years indicating its central importance to cardiovascular disease and was included in the recent National Cholesterol Educational Program – Adult Treatment Panel III (NCEP ATP III) clinical guidelines in order to assist the clinician in using this important tool to evaluate additional cardiovascular risk [16-19].

### Hyperinsulinemia and Hyperamylinemia

Insulin, proinsulin, and amylin individually and synergistically activate the renin – angiotensin system (RAS) with subsequent increase in Ang II. Ang II is the most potent endogenous inducer of NAD(P)H oxidase, increasing NAD(P)H, which increases vascular – intimal reactive oxygen species (ROS) and superoxide (O_2_^-•^) [19,20]. There are many deleterious effects of hyperinsulinemia in addition to its being responsible for sodium, potassium, water, and urate retention in proximal kidney (table 3) [21].

**Table 3:** Deleterious effects of hyperinsulinemia (HI)

**1.**	HI, hyperproinsulinemia, and hyperamylinemia synergistically activate RAS with subsequent increase in Ang II, renin, and aldosterone.
**2.**	HI promotes Na^+ ^and H_2_O retention, which increases blood volume and pressure. In turn this activates the reabsorption of uric acid resulting in elevation of SUA. In turn increased SUA has been shown to increase tubular reabsorption of Na+.
**3.**	HI increases membrane cation-transport increasing intracellular Ca^++^, which increases tone and pressure.
**4.**	HI activates the sympathetic nervous system.
**5.**	HI stimulates vSMC proliferation and migration and remodeling.
**6.**	HI increases the number of AT-1 receptors.
**7.**	HI creates cross talk between the insulin receptor and AT-1 receptor, resulting in a more profound Ang II effect.
**8**	HI promotes PI3 kinase Akt-MAP kinase Shunt. Impairing the metabolic (PI3 kinase-AKT pathway while promoting the MAPkinase remodeling pathway.
**9.**	HI induces Ang II, which promotes the MAP kinase pathway and remodeling.
**10.**	HI induces Ang II, which is the most potent stimulus for production of NAD(P)H oxidase with reactive oxygen species generation (superoxide production) and resultant vascular oxidative stress.

### Hypertension

Hypertension is strongly associated with hyperuricemia. SUA levels are elevated in hypertension and are present in 25% of untreated hypertensive subjects, 50% of subjects taking diuretics, and greater than 75% of patients with malignant hypertension [22]. Potential mechanisms involved with the association of hyperuricemia and hypertension include the following: 1. Decreased renal blood flow (decreased GFR) stimulating urate reabsorption, 2. Microvascular (capillary) disease resulting in local tissue ischemia. 3. Ischemia with associated increased lactate production that blocks urate secretion in the proximal tubule and increased uric acid synthesis due to increased RNA-DNA breakdown and increased purine (adenine and guanine) metabolism, which increases uric acid and ROS through the effect of xanthine oxidase (XO). 4. Ischemia induces increased XO production and increased SUA and ROS. These associations with ischemia and XO induction may help to understand why hyperuricemia is associated with preeclampsia and congestive heart failure.

Because endothelial dysfunction, local oxidant generation, elevated circulating cytokines, and a proinflammatory state are common in patients with cardiovascular disease and hypertension there is an increased level of oxidative – redox stress within vascular tissues. Oxidative – redox stress results in impaired endothelium-dependent vasodilation with quenching of endothelial nitric oxide (eNO) and allows the endothelium to become a net producer of ROS specifically superoxide as the endothelial nitric oxide synthase (eNOS) enzyme uncouples to produce superoxide instead of eNO. This similar mechanism applies equally well to that associated with type 2 diabetes and congestive heart failure [11,19]. It is important to note that allopurinol and oxypurinol (XO inhibitors) are capable of reversing the impaired eNO production in both heart failure [23-25] and type 2 diabetes mellitus (T2DM) [26].

Lin KC *et al*. were able to demonstrate that blood pressure levels were predictive for cardiovascular disease incidence synergistically with serum uric acid level [27]. Two separate laboratories have demonstrated the development of systemic hypertension in a rat model of hyperuricemia developed with a uricase inhibitor (oxonic acid) after several weeks of treatment [28,29]. This hypertension was associated with increased renin and a decrease in neuronal nitric oxide synthase in the juxtaglomerular apparatus. Prevention of this hypertension was accomplished by an ACE inhibitor and to a lesser extent L-arginine. These findings indicate an interacting role of the renin- angiotensin system and the NOS enzyme. Hypertension, neural nitric oxide synthase (nNOS) and renin changes were also prevented by maintaining uric acid levels in the normal range with allopurinol or benziodarone (a uricosuric).

These above models have provided the first challenging evidence that uric acid may have a pathogenic role in the development of hypertension, vascular disease, and renal disease [11].

### Obesity

**Figure 1:**
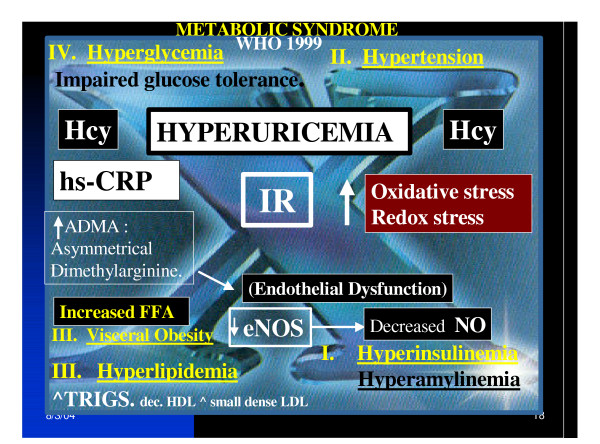
**Metabolic syndrome: hyperuricemia. **This image focuses on the "H" phenomenon consisting of the four major players in the MS: Hyperinsulinemia, Hypertension, Hyperlipidemia and the Lipotoxicity – Obesity toxicity triad, and Hyperglycemia. These players have frequently been referred to as the "deadly quartet" and the "H" phenomenon. It is important to note the central position of insulin resistance in this image and also hyperuricemia. Hyperuricemia is flanked by hyperhomocysteinemia to indicate its importance in the MS. Each of these players has its own important role and this image helps to portray the clustering effect and synergism to contribute to an overall increased oxidative – redox stress to the endothelium of the vasculature.

Obesity has reached epidemic proportions in the past decade and represents one of the confounding factors associated with the MS and T2DM [19,30] (figure 1).

Hyperuricemia has been associated with increasing body mass index (BMI) in recent studies and are even apparent in the adolescent youth [30-33].

Leptin levels are elevated and associated with insulin resistance in MS and early T2DM. Bedir A *et al. *have recently discussed the role of leptin as possibly being a regulator of SUA concentrations in humans and even suggested that leptin might be one of the possible candidates for the missing link between obesity and hyperuricemia [34]. Furthermore, hypertriglyceridemia and free fatty acids are related to hyperuricemia independently of obesity and central body fat distribution [30,33] (table 1: (T): Triglyceride toxicity and (F): Free fatty acid toxicity).

### Hyperglycemia: Impaired glucose tolerance: Type 2 Daibetes Mellitus (T2DM)

Glucotoxicity places an additional burden of redox stress on the arterial vessel wall and capillary endothelium. Hyperglycemia induces both an oxidative stress (glucose autoxidation and advanced glycosylation endproducts (AGE) – ROS oxidation products) and a reductive stress through pseudohypoxia with the accumulation of NADH and NAD(P)H in the vascular intima [19,35,36].

This redox stress consumes the natural occurring local antioxidants such as: SOD, GPX, and catalase (table 4). Once these local intimal antioxidants are depleted uric acid can undergo the paradoxical antioxidant – prooxidant switch or the urate redox shuttle [37,38]

**Table 4:** Antioxidants: enzymatic – nonenzymatic inactivation of free radicals.

**ENZYMATIC ANTIOXIDANTS**
SUPER OXIDE DISMUTASE (SOD) Reactions catalyzed: [O_2_^- ^+ SOD → H_2_O_2 _+ O_2_] Various isoforms: ecSOD (extracellular); Mn-SOD (mitochondrial); Cu/Zn-SOD (intracellular)
CATALASE – Location: peroxisome. Reaction catalyzed: [2 H_2_O_2 _+ catalase → 2 H_2_O + O_2_]
GLUTATHIONE PEROXIDASE – Location: mitochondrion, cytosol, and systemic circulation. Glutathione (GSH or glutamyl-cysteinyl-glycine tripeptide): the reduced -SH of GSH is oxidized to disulfide GSSG. Glutathione peroxidase-catalyzed reation: [GSH + 2 H_2_O_2 _→ GSSG + H_2_O + O_2_] Glutathione reductase-catalyzed reaction: [GSSG → GSH] at the expense of [NADH → NAD^+^] and/or [NAD(P)H → NAD(P)^+^]
**ENZYMATIC – NONENZYMATIC INACTIVATION OF FREE RADICALS. NITRIC OXIDE SYNTHASE **Location: membrane.
Isoforms:
eNOS (endothelial): good
nNOS (neuronal): good
iNOS (inducible-inflammatory): bad
O_2_^- ^and nitric oxide (NO) are consumed in this process with the creation of reactive nitrogen species (RNS). O_2_^- ^+ NO → ONOO-(peroxynitrite) + tyrosine → nitrotyrosine. Nitrotyrosine reflects redox stress and leaves a measurable footprint. NO the good; O_2_^• ^the bad; ONOO^- ^the ugly *
**NONENZYMATIC ANTIOXIDANTS**
Vitamins (A, C, and E): Thiols: Sulfhydryl (-SH)-containing molecules. Albumin: Is an antioxidant because of it is a thiol-containing macromolecule. Apoproteins: Ceruloplasmin and transferrin. Bind copper and iron in forms, which cannot participate in the Fenton reaction. **Uric acid:**Early on in the atherosclerotic process in physiologic ranges: antioxidant. **PARADOX:**Late in elevated range prooxidant with loss of supporting antioxidants above and in a milieu of oxidative – redox stress within the atherosclerotic intima. In MS, T2DM and advanced vulnerable atherosclerotic plaques SOD, Catalase, and GPX are depleted. **The Urate Redox Shuttle**. **PARADOX: **antioxidants may become prooxidant in a certain milieu.

* Beckman JS and Koppenol WH [1996] Nitric oxide, superoxide, and peroxynitrite: the good, the bad, and ugly. Am J Physiol 271(5 Part 1): C1424–C1437

### Homocysteine

A direct relation between homocysteine levels and SUA levels is known to occur in patients with atherosclerosis. Not only do these two track together (possibly reflecting an underlying elevated tension of redox stress) but also may be synergistic in creating an elevated tension of redox stress, especially in the rupture prone, vulnerable atherosclerotic plaque with depletion of local occurring antioxidants [39-41] (figure 1).

### Atherosclerosis and Atheroscleropathy

Non-diabetic atherosclerosis and atheroscleropathy (accelerated atherosclerosis associated with MS, prediabetes, and T2DM) are each impacted with the elevation of uric acid [42,43].

### Prothrombotic milieu

In MS and T2DM there is an observed increased thrombogenecity, hyperactive platelets, increased PAI-1 (resulting in impaired fibrinolysis), and increased fibrinogen in the atherosclerotic milieu associated with the dysfunctional endothelial cell. Additionally, the vulnerable atherosclerotic plaque includes increased tissue factor, which increases the potential for thrombus formation when the plaque ruptures and exposes its contents to the lumen [19,42,43].

## Uric acid as one of the multiple injurious stimuli to the endothelium of the arterial vessel wall and capillary

**Figure 2:**
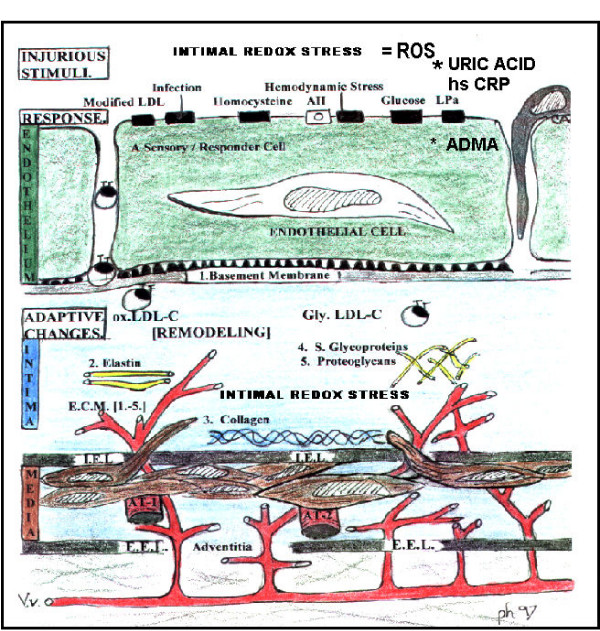
**Multiple injurious stimuli to the endothelium in non-diabetic atherosclerosis and atheroscleropathy. **This image portrays the anatomical relationship between the endothelium, intima, media and the adventitia. Each of these layers plays an important role in the development of accelerated atherosclerosis (atheroscleropathy) of the MS, PD, and overt T2DM. Of all the different layers the endothelium seems to play a critical and central role. It is placed at a critical location and acts as an interface with nutrients and toxic products not only at its luminal surface of musculo-elastic arteries but also at the endothelial extracellular matrix interface of the interstitium in capillary beds. The intima, sandwiched between the medial muscular layer and the endothelium, is the site of atherosclerosis, intimopathy, and the atheroscleropathy associated with MS, PD, and overt T2DM. There are multiple injurious stimuli to the endothelium including ROS and hyperuricemia. It is important to note that redox stress occurs upstream from inflammation by activating the nuclear transcription factor: NFkappa B [39]. Over time, individually and synergistically these injurious stimuli (table 1) result in the morbid – mortal vascular complications of MS, T2DM, atheroscleropathy, and non-diabetic atherosclerosis.

The upper 1/3 of the normal physiologic – homeostatic range (> 4 mg/dl) and abnormal elevations (> 6.5 or 7 mg/dl in men and > 6.0 mg/dl in women) in SUA definitely should be considered as one of the multiple injurious stimuli to the arterial vessel wall and capillary, which may contribute to endothelial dysfunction and arterial – capillary vessel wall remodeling through oxidative – redox stress [2,3,19] (figure 2). There are multiple injurious stimuli to the endothelium and arterial vessel wall in the accelerated atherosclerosis associated with MS and T2DM (atheroscleropathy)(figure 2). It is important to note that redox stress occurs upstream from inflammation by activating the nuclear transcription factor: NFkappa B [39]. Over time, individually and synergistically injurious stimuli of the A-FLIGHT-U acronym (table 1) result in the morbid – mortal complications of MS, T2DM, atheroscleropathy, and non-diabetic atherosclerosis.

Each of these A-FLIGHT-U toxicities may be viewed as an independent risk marker – factor and is known to have a synergistic effect when acting in concert [19,21,39,42,43]. Additionally, low density lipoproteins such as LDL-cholesterol are capable of being modified and retained within the intima through a process of oxidative modification through free radicals, hypochlorous acid, peroxynitrite, and selected oxidative enzymes such as xanthine oxidase, myeloperoxidase and lipoxygenase (table 5) [19,44-50].

**Table 5:** Origin, enzymatic pathways of reactive oxygen species, and their oxidized products.

[Origin and Location] Enzymatic Pathways:	[ROS] Potent Oxidants:	[Products] Oxidized lipids and proteins:
Mitochondrial Respiratory Chain	O_2_^•^ -OH^•^	Oxidized lipids, proteins, nucleic acids, and autoxidation byproducts
Inflammatory Macrophage Membranous NAD(P)H Oxidase	O_2_^•^ -OH^•^ H_2_O_2_	Advanced lipoxidation endproducts (ALE) *ortho *o-tyrosine *meta *m-tyrosine
Granular Myeloperoxidase (MPO)	Hypochlorous acid HOCL Tyr (Tyrosine) NO_2_	3-Chlorotyrosine di-Tyrosine NO_2_^-^(Nitrotyrosine)
Macrophage		
Nitric Oxide Synthase (iNOS) Inducible (iNOS) Large bursts – uncontrolled	ONOO^•^	NO_2_^-^(Nitrotyrosine)
Endothelial Cell		
Nitric Oxide Synthase (NOS) Constitutive (cNOS) eNOS → NO nNOS → NO Small bursts – controlled	NO + **O_2_^• ^**→ ONOO^•^ ONOO^•^	NO_2_^-^(Nitrotyrosine) NO_2_^-^(Nitrotyrosine)
eNOS-derived NO	**NO **The GOOD *	Natural-occurring, local-occurring, chain-breaking, antioxidant
Superoxide	**O_2_^• ^**The BAD *	Toxic effects of ROS on proteins, lipid, nucleic acids
Peroxynitrite	**ONOO^• ^**The UGLY *	Toxic effects of ROS on proteins, lipid, nucleic acids
Hypochlorous acid	**HCLO **The UGLY *	Toxic effects of ROS on proteins, lipid, nucleic acids
Restoration of eNO Via the eNOS reaction	Antioxidant Antioxidant	Prevention of the toxic effects of ROS

* Beckman JS and Koppenol WH [1996] Nitric oxide, superoxide, and peroxynitrite: the good, the bad, and ugly. Am J Physiol 271(5 Part 1): C1424–C1437

The simple concept that SUA in patients with CVD, MS, T2DM, hypertension, and renal disease may reflect a compensatory mechanism to counter oxidative stress is intriguing. However, this does not explain why higher SUA levels in patients with these diseases are generally associated with worse outcomes [11].

### An antioxidant – prooxidant urate redox shuttle

**Figure 3:**
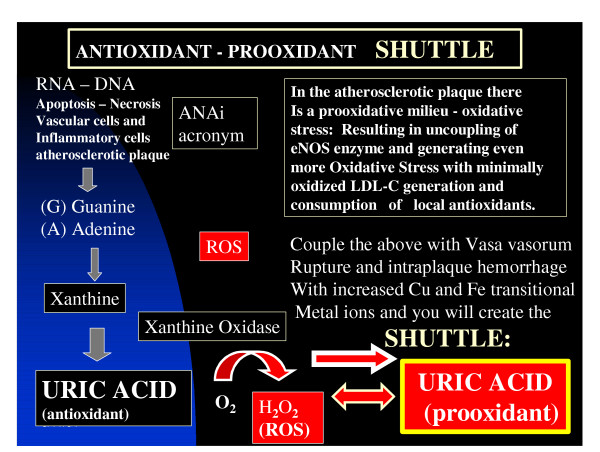
**Antioxidant – prooxidant urate redox shuttle. **The antioxidant – prooxidant urate redox shuttle is an important concept to understand regarding accelerated atherosclerosis. This shuttle is important in understanding the role of how the antioxidant uric acid becomes prooxidant in this environmental milieu, which results in its damaging role to the endothelium and arterial vessel wall remodeling with an elevated tension of oxidative – redox stress (ROS), accelerated atherosclerosis and arterial vessel wall remodeling.

Antioxidants may become prooxidants in certain situations [51-55]. Therefore we propose the existence of an antioxidant – prooxidant redox shuttle in the vascular milieu of the atherosclerotic macrovessel intima and the local sub endothelial capillary interstitium of the microvessel [38,51,52] (figure 3).

SUA in the early stages of the atherosclerotic process is known to act as an antioxidant and may be one of the strongest determinates of plasma antioxidative capacity [53].

**Figure 4:**
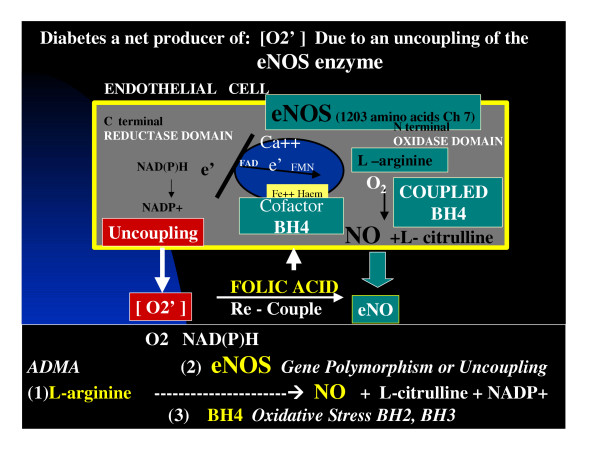
**Uncoupling of the eNOS reaction. **It is important to understand the role of endothelial dysfunction in accelerated atherosclerosis and even more important to understand the role of eNOS enzyme uncoupling and how it relates to MS, PD, T2DM, and non-diabetic atherosclerosis. Oxygen reacts with the eNOS enzyme in which the tetrahydrobiopertin (BH_4_) cofactor has coupled nicotinamide dinucleotide phosphate reduced (NAD(P)H) emzyme with L-arginine to be converted to nitric oxide (NO) and L-citrulline. When uncoupling occurs the NAD(P)H enzyme reacts with O_2 _and the endothelial cell becomes a net producer of superoxide (O_2_^•^) instead of the protective endothelial NO. This figure demonstrates the additional redox stress placed upon the arterial vessel wall and capillaries in patients with MS, PD, and overt T2DM.

However, later in the atherosclerotic process when SUA levels are known to be elevated (in the upper 1/3 of the normal range >4 mg/dl and outside of the normal range >6 mg/dl in females and 6.5–7 mg/dl in males) this previously antioxidant (SUA) paradoxically becomes prooxidant. This antioxidant – prooxidant urate redox shuttle seems to rely heavily on its surrounding environment such as timing (early or late in the disease process), location of the tissue and substrate, acidity (acidic – basic – or neutral ph), the surrounding oxidant milieu, depletion of other local antioxidants, the supply and duration of oxidant substrate and its oxidant enzyme. In the accelerated atherosclerotic – vulnerable plaque the intima has been shown to be acidic [54], depleted of local antioxidants with an underlying increase in oxidant stress and ROS (table 1) (table 5) and associated with uncoupling of the eNOS enzyme and a decrease in the locally produced naturally occurring antioxidant: eNO and endothelial dysfunction. This process is also occurring within the microvascular bed at the level of the capillary within various affected hypertensive and diabetic end organs [19,51,52] (figure 4).

Nitric oxide and vitamin C have each been shown to inhibit the prooxidant actions of uric acid during copper-mediated LDL-C oxidation [38,55].

### The ANAi acronym

We have devised an acronym, to better understand the increase in SUA synthesis within the accelerated atherosclerotic plaque termed: ANAi. A – apoptosis, N – necrosis, A – acidic atherosclerotic plaque, angiogenesis (both induced by excessive redox stress), i – inflammation, intraplaque hemorrhage increasing red blood cells – iron and copper transition metal ions within the plaque.

This acronym describes the excess production of purines: (A) adenine and (G) guanine base pairs from RNA and DNA breakdown due to apoptosis and necrosis of vascular cells in the vulnerable – accelerated atherosclerotic plaques; allowing SUA to undergo the antioxidant – prooxidant urate redox shuttle (figure 3).

Reactions involving transitional metal ions such as copper and iron are important to the oxidative stress within atherosclerotic plaques. Reactions such as the Fenton and Haber- Weiss reactions and similar reactions with copper lead to an elevated tension of oxidative – redox stress.

FENTON REACTION:

Fe^2+ ^+ H_2_O_2 _→ Fe^3+ ^+ OH^• ^+ OH^-^

Fe^3+ ^+ H_2_O_2 _→ Fe^2+ ^+ OOH^• ^+ H^+^

HABER – WEISS REACTION:

H_2_O_2 _+ O_2_^- ^→ O2 + OH^- ^+ OH

H_2_O_2 _+ OH^- ^→ H_2_O + O_2_^- ^+ H^+^

The hydroxyl radicals can then proceed to undergo further reactions with the production of ROS through addition reactions, hydrogen abstraction, electron transfer, and radical interactions. Additionally, copper (Cu^3+ ^- Cu^2+ ^- Cu^1+^) metal ions can undergo similar reactions with formation of lipid peroxides and ROS. This makes the leakage of iron and copper from ruptured vasa vasorum very important in accelerating oxidative damage to the vulnerable accelerated atherosclerotic plaques, as well as, providing a milieu to induce the SUA antioxidant – prooxidant switch within these plaques [42].

These same accelerated – vulnerable plaques now have the increased substrate of SUA through apoptosis and necrosis of vascular cells (endothelial and vascular smooth muscle cells) and the inflammatory cells (primarily the macrophage and to a lesser extent the lymphocyte).

### Endothelial function and endothelial nitric oxide (eNO)

The endothelium is an elegant symphony responsible for the synthesis and secretion of several biologically active molecules. It is responsible for regulation of vascular tone, inflammation, lipid metabolism, vessel growth (angiogenesis – arteriogenesis), arterial vessel wall – capillary sub endothelial matrix remodeling, and modulation of coagulation and fibrinolysis. One particular enzyme system seems to act as the maestro: The endothelial nitric oxide synthase (eNOS) enzyme and its omnipotent product: endothelial nitric oxide (eNO) (figure 2).

The endothelial nitric oxide synthase (eNOS) enzyme reaction is of utmost importance to the normal functioning of the endothelial cell and the intimal interstitium. When this enzyme system uncouples the endothelium becomes a net producer of superoxide and ROS instead of the net production of the protective antioxidant properties of eNO (table 6) (figure 4).

**Table 6:** The positive effects of eNOS and eNO

• Promotes vasodilatation of vascular smooth muscle.
• Counteracts smooth muscle cell proliferation.
• Decreases platelet adhesiveness.
• Decreases adhesiveness of the endothelial layer to monocytic WBCs (the "teflon effect").
• Anti-inflammatory effect.
• Anti-oxidant effect. It scavenges reactive oxygen species locally, and acts as a chain-breaking antioxidant to scavenge ROS.
• Anti-fibrotic effect. When NO is normal or elevated, MMPs are quiescent; conversely if NO is low, MMPs are elevated and active.
MMPs are redox sensitive.
• No inhibits prooxidant actions of uric acid during copper-mediated LDL oxidation.
• NO has diverse anti-atherosclerotic actions on the arterial vessel wall including antioxidant effects by direct scavenging of ROS – RNS acting as chain-breaking antioxidants and it also has anti-inflammatory effects.

There are multiple causes for endothelial uncoupling in addition to hyperuricemia and the antioxidant – prooxidant urate redox shuttle: A-FLIGHT -U toxicities, ROS, T2DM, prediabetes, T1DM, insulin resistance, MS, renin angiotensin aldosterone activation, angiotensin II, hypertension, endothelin, dyslipidemia – hyperlipidemia, homocysteine, and asymmetrical dimethyl arginine (ADMA) [19,39,43].

Xanthine oxidase – oxioreductase (XO) has been shown to localize immunohistochemically within atherosclerotic plaques allowing the endothelial cell to be equipped with the proper machinery to undergo active purine metabolism at the plasma membrane surface, as well as, within the cytoplasm and is therefore capable of overproducing uric acid while at the same time generating excessive and detrimental ROS [56] (figure 3,4). To summarize this section:

The healthy endothelium is a net producer of endothelial nitric oxide (eNO).

The activated, dysfunctional endothelium is a net producer of superoxide (O_2_^-^) associated with MS, T2DM, and atheroscleropathy [43].

## Uric acid and inflammation

Uric acid and highly sensitive C reactive protein (hsCRP) each now share a respected inclusion as two of the novel risk markers – risk factors associated with the metabolic syndrome. It is not surprising that these two markers of risk track together within the MS. If there is increased apoptosis and necrosis of vascular cells and inflammatory cells in accelerated – vulnerable atherosclerotic plaques as noted in the above section then one would expect to see an increase in the metabolic breakdown products of RNA and DNA with arginine and guanine to its end product of uric acid. SUA elevation may indeed be a sensitive marker for underlying vascular inflammation and remodeling within the arterial vessel wall and capillary interstitium.

Is it possible that SUA levels could be as similarly predictive as hsCRP since it is a sensitive marker for underlying inflammation and remodeling within the arterial vessel wall and the myocardium [57].

Should the measurement of SUA be part of the national cholesterol educational program adult treatment panel III and future IV (NCEP ATPIII or the future NCEP ATPIV) clinical guidelines (especially in certain ethnic groups such as females and in the African Americans)?

Uric acid is known to induce the nuclear transcription factor (NF-kappaB) and monocyte chemoattractant protein-1 (MCP-1) [58]. Regarding TNF alpha it has been shown that SUA levels significantly correlate with TNF alpha concentrations in congestive heart failure and as a result Olexa P *et al*. conclude that SUA may reflect the severity of systolic dysfunction and the activation of an inflammatory reaction in patients with congestive heart failure [59]. Additionally, uric acid also stimulates human mononuclear cells to produce interleukin-1 beta, IL-6, and TNF alpha [11].

Tamakoshi K *et al*. have shown a statistically significant positive correlation between CRP and body mass index (BMI), total cholesterol, triglycerides, LDL-C, fasting glucose, fasting insulin, uric acid, systolic blood pressure, and diastolic blood pressure and a significant negative correlation of CRP with HDL-C in a study of 3692 Japanese men aged 34–69 years of age. They conclude that there are a variety of components of the MS, which are associated with elevated CRP levels in a systemic low-grade inflammatory state [60].

CRP and IL-6 are important confounders in the relationship between SUA and overall mortality in elderly persons, thus when evaluating this association the potential confounding effect of underlying inflammation and other risk factors should be considered [61].

## Uric acid and chronic renal disease

Hyperuricemia can be the consequence of increased uric acid production or decreased excretion. Any cause for decreased glomerular filtration, tubular excretion or increased reabsorption would result in an elevated SUA. Increased SUA has been found to predict the development of renal insufficiency in individuals with normal renal function [11]. In T2DM hyperuricemia seems to be associated with MS and with early onset or increased progression to overt nephropathy, whereas hypouricemia was associated with hyperfiltration, and a later onset or decreased progression to overt nephropathy [62]. An elevated SUA could be advantageous information for the clinician when examining the global picture of T2DM in order to detect those patients who might gain from more aggressive global risk reduction to delay or prevent the transition to overt nephropathy. Elevated SUA contributes to endothelial dysfunction and increased oxidative stress within the glomerulus and the tubulo-interstitium with associated increased remodeling fibrosis of the kidney and as noted earlier in this discussion to be pro-atherosclerotic and proinflammatory. This would have a direct effect on the vascular supply affecting macrovessels, particularly the afferent arterioles. The glomeruli would be affected also through the effect of uric acid on the glomerular endothelium with endothelial dysfunction due to oxidative – redox stress and result in glomerular remodeling. SUA's effect on hypertension would have an additional affect on the glomeruli and the tubulo-interstitium with remodeling changes and progressive deterioration of renal function. Increased ischemia – ischemia reperfusion would activate the xanthine oxidase mechanism and contribute to an increased production of ROS through H_2_O_2 _generation and oxidative stress within the renal architecture with resultant increased remodeling. Hyperuricemia could increase the potential for urate crystal formation and in addition to elevated levels of soluble uric acid could induce inflammatory and remodeling changes within the medullary tubulo-interstitium.

A recent publication by Hsu SP *et al. *revealed a J-shaped curve association with SUA levels and all-cause mortality in hemodialysis patients [63]. They were able to demonstrate that decreased serum albumin, underlying diabetic nephropathy, and those in the lowest and highest quintiles of SUA had higher all-cause mortality. It is interesting to note that almost all of the large trials with SUA and cardiovascular events have demonstrated this same J shaped curve regarding all-cause mortality with the nadir of risk occurring in the second quartile [11].

Johnson RJ *et al*. have speculated that the increased risk for the lowest quartile reflects a decreased antioxidant activity, while the increased risk at higher levels reflects the role of uric acid in inducing vascular disease and hypertension through the mechanism of the previously discussed antioxidant prooxidant urate redox shuttle. This would suggest that treatment with xanthine oxidase inhibitors (allopurinol) should strive to bring levels to the 3–4 mg/dl range and not go lower [11].

## Nutritional support for hyperuricemia

While it is not within the scope of this review to discuss this important topic with an in- depth examination, it is important to discuss some prevailing concepts and provide some clinical nutritional guidelines for hyperuricemia (table 8).

**Table 8:** Nutritional guidelines for hyperuricemia

**Obesity**	Caloric restriction to induce weight loss in order to decrease insulin resistance of the MS. Exercise to aid in weight reduction by increased energy expenditure, also to increase eNOS and eNO, as well as, increase HDL-C with its antioxidant – anti-inflammatory effects. Both will result in **REDOX STRESS REDUCTION**
**Alcohol**	Avoidance and or moderation. Especially beer with the increased purines from hops and barley. Also improve the liver antioxidant potential. **REDOX STRESS REDUCTION**
**Low purine diet (moderation)**	Moderation in meats and seafood's, especially shrimp and barbeque ribs (all you can eat specials). Vegetables and fruits higher in purine should not be completely avoided as they provide fiber and naturally occurring antioxidants. Lists should be provided to demonstrate the vegetables and fruits that are higher in purines to allow patients healthier choices **REDOX STRESS REDUCTION**
**Fiber**	Emphasize the importance of fiber in the diet as fiber helps to bind excess purines in the gastrointestinal track. **REDOX STRESS REDUCTION**

Moderation is the key element in any diet approaching hyperuricemia. The nutritional "gold standard" for the treatment of hyperuricemia has been "the low purine diet". This traditional diet has recently come into question as it may limit the intake of high purine vegetables and fruits. Vegetables and fruits are important for the fiber they supply in addition to naturally occurring antioxidants. Recently, of greater importance is controlling obesity through generalized caloric restriction and increased exercise to combat the overnutrition and underexercise of our modern-day society, as well as, controlling the consumption of alcohol [64].

Nutritional support by the nutritionist and the diabetic educator (an integral part of the health care team) is of utmost importance when dealing with the metabolic syndrome, T2DM, and the cardiovascular atherosclerotic afflicted patients in order to obtain global risk reduction, because we are what we eat.

## Conclusion

From a clinical standpoint, hyperuricemia should alert the clinician to an overall increased risk of cardiovascular disease and especially those patients with an increased risk of cardiovascular events. Hyperuricemia should therefore be a "red flag" to the clinician to utilize a team effort in achieving an overall approach to obtain a global risk reduction program through the use of the RAAS acronym (table 7).

**Table 7:** The RAAS Acronym: GLOBAL RISK REDUCTION

**R**	**Reductase inhibitors **(HMG-CoA). Decreasing modified LDL-cholesterol, i.e., oxidized, acetylated LDL-cholesterol. Decreasing triglycerides and increasing HDL-cholesterol. Improving endothelial cell dysfunction. Restoring the abnormal Lipoprotein fractions. Thus, decreasing the redox and oxidative stress to the arterial vessel wall and myocardium.
	***Redox stress reduction***
**A**	AngII inhibition or receptor blockade: **ACEi-prils. ARBs-sartans. **Both inhibiting the effect of angiotensin-II locally as well as systemically. Affecting hemodynamic stress through their antihypertensive effect as well as the deleterious effects of angiotensin II on cells at the local level – injurious stimuli -decreasing the stimulus for O_2_^• ^production. Decreasing the A-FLIGHT toxicities. The positive effects on microalbuminuia and delaying the progression to end stage renal disease. Plus the direct-indirect antioxidant effect within the arterial vessel wall and capillary. Antioxidant effects. **Aspirin **antiplatelet, anti-inflammatory effect on the diabetic hyperactive platelet. **Adrenergic (non-selective blockade) **in addition to its blockade of prorenin → renin conversion. **Amlodipine **– Felodipine with calcium channel blocking antihypertensive effect, in addition to their direct antioxidant effects.
	***Redox stress reduction***
**A**	**Aggressive control of diabetes **to HbA1c of less than 7. This usually requires combination therapy with the use of insulin secretagogues, insulin sensitizers (PPAR-gamma agonists), biguanides, alpha-glucosidase inhibitors, and ultimately exogenous insulin. Decreasing modified LDL cholesterol, i.e., glycated-glycoxidated LDL cholesterol. Improving endothelial cell dysfunction. Also decreasing glucotoxicity and the oxidative-redox stress to the intima and pancreatic islet. **Aggressive control of blood pressure**, which usually requires combination therapy, including thiazide diuretics to attain JNC 7 guidelines. **Aggressive control of homocysteine **with folic acid with its associated additional positive effect on re-coupling the eNOS enzyme reaction by restoring the activity of the BH_4 _cofactor to run the eNOS reaction via a folate shuttle mechanism and once again produce eNO. **Aggressive control of uric acid **levels with xanthine oxidase inhibitors (allopurinol and oxypurinol) should be strongly considered in view of the prevailing literature in order to achieve more complete: Global Risk Reduction
	***Redox stress reduction***
**S**	**Statins. **Improving plaque stability (pleiotropic effects) independent of cholesterol lowering. Improving endothelial cell dysfunction. Moreover, the direct/indirect antioxidant anti-inflammatory effects within the islet and the arterial vessel wall promoting stabilization of the unstable, vulnerable islet and the arterial vessel wall. **Style. **Lifestyle modification (weight loss, exercise, and change eating habits). **Stop Smoking.**
	***Redox stress reduction***

SUA may or may not be an independent risk factor especially since its linkage to other risk factors is so strong, however there is not much controversy regarding its role as a marker of risk, or that it is clinically significant and relevant.

Regarding the MS and epidemiologic evaluations: A multivariate model could well eliminate hyperuricemia as an independent risk factor even if it were contributing to the overall phenotypic risk of the syndrome. Additionally, we must remember that it was Reaven that called for the inclusion of hyperuricemia to Syndrome X we now call MS – insulin resistance syndrome -IRS in 1993 [18].

A quote by Johnson RJ and Tuttle KR is appropriate for the concluding remarks:

"The bottom line is that measuring uric acid is a useful test for the clinician, as it carries important prognostic information. An elevation of uric acid is associated with an increased risk for cardiovascular disease and mortality, especially in women" [64].

## Abbreviations

Serum uric acid (SUA); cardiovascular disease (CVD); coronary heart disease (CHD); endothelial nitric oxide (eNO); endothelial nitric oxide synthase (eNOS); endothelial nitric oxide (eNO); reactive oxygen species (ROS); metabolic syndrome (MS); insulin resistance syndrome (IRS); nicotine adenine dinucleotide phosphate oxidase reduced NAD(P)H; superoxide (O_2_^-•^); xanthine oxidase (XO); type 2 diabetes mellitus (T2DM); angiotensin converting enzyme (ACE); renin-angiotensin-aldosterone system (RAAS); advanced glycosylation endproducts (AGE); superoxide dismutase (SOD); glutathione (GPX); plasminogen activator inhibitor (PAI-1); angiotensin II (AngII); low density lipoprotein cholesterol (LDL-C); asymmetrical dimethyl arginine (ADMA); highly sensitive C reactive protein (hsCRP); national cholesterol educational program adult treatment panel III (NCEP ATPIII); nuclear transcription factor (NF-kappaB); monocyte chemoattractant protein-1 (MCP-1); tumor necrosis factor alpha (TNF alpha); interleukin one beta (IL-1beta); interleukin 6 (IL-6); body mass index (BMI); high density lipoprotein (HDL); hydrogen peroxide (H_2_O_2_); free fatty acids (FFA).

## Competing interests

The authors declare that they have no competing interests.

## Author's contribtions

MRH and SCT envisioned, wrote and edited jointly.
